# Oxygen consumption in seasonally stratified lakes decreases only below a marginal phosphorus threshold

**DOI:** 10.1038/s41598-019-54486-3

**Published:** 2019-12-02

**Authors:** Beat Müller, Thomas Steinsberger, Robert Schwefel, René Gächter, Michael Sturm, Alfred Wüest

**Affiliations:** 1 Eawag, Swiss Federal Institute of Aquatic Science and Technology, Surface Waters – Research and Management, Kastanienbaum, Switzerland; 2 Physics of Aquatic Systems Laboratory, Margaretha Kamprad Chair, ENAC-IEE-APHYS, École Polytechnique Fédérale de Lausanne (EPFL), Lausanne, Switzerland; 3 Present Address: UC Santa Barbara, 3015 Marine Science Building, Santa Barbara, CA 93106-6150 USA

**Keywords:** Limnology, Biogeochemistry

## Abstract

Areal oxygen (O_2_) consumption in deeper layers of stratified lakes and reservoirs depends on the amount of settling organic matter. As phosphorus (P) limits primary production in most lakes, protective and remediation efforts often seek to reduce P input. However, lower P concentrations do not always lead to lower O_2_ consumption rates. This study used a large hydrochemical dataset to show that hypolimnetic O_2_ consumption rates in seasonally stratified European lakes remain consistently elevated within a narrow range (1.06 ± 0.08 g O_2_ m^−2^ d^−1^) as long as areal P supply (APS) exceeded 0.54 ± 0.06 g P m^−2^ during the productive season. APS consists of the sum of total P present in the productive top 15 m of the water column after winter mixing plus the load of total dissolved P imported during the stratified season, normalized to the lake area. Only when APS sank below this threshold, the areal hypolimnetic mineralization rate (AHM) decreased in proportion to APS. Sediment trap material showed increasing carbon:phosphorus (C:P) ratios in settling particulate matter when APS declined. This suggests that a decreasing P load results in lower P concentration but not necessarily in lower AHM rates because the phytoplankton community is able to maintain maximum biomass production by counteracting the decreasing P supply by a more efficient P utilization. In other words, in-lake organic matter production depends only on APS if the latter falls below the threshold of 0.54 g P m^−2^ and correspondingly, the atomic C:P ratio of the settling material exceeds ~155.

## Introduction

Since the 1970s, consensus on lake biogeochemistry has held that after light levels, P input exerts the strongest influence on primary production and by extension, trophic state^1–3^. This relation implies that primary production and hence phytoplankton density increases with P supply until maximum plankton density is constrained mostly due to self-shading in the water column^4^. Above this point, additional P no longer enhances organic matter (OM) production but instead results in excessive P accumulation within OM. This in turn leads to lower C:P ratios in the suspended and settling particles^5–9^.

Excessive P input to lakes, reservoirs, and coastal marine ecosystems is thus interpreted to stimulate phytoplankton growth in their surface layer and subsequent O_2_ depletion in deep waters where resulting anoxic conditions can potentially endanger aquatic life^10–12^. Mitigation strategies therefore typically focus on i) restriction of external P input, ii) in-lake P reduction^13^, and iii) aeration of O_2_-depleted water layers^14–16^. Research has shown that lowering external P loads results in lower P concentrations^3,17^ but not necessarily in lower hypolimnetic O_2_ consumption rates^18^.

Almost a century of research has addressed hypolimnetic O_2_ consumption^19–24^. Primary O_2_ sinks are (i) respiration and mineralization of OM settling through the water column^25^, (ii) the sediment surface, where settled OM is deposited and further processed, although at a potentially slower rate^18^, and iii) the lowermost water column layers, where oxidation of reduced sediment-borne compounds (e.g., methane, ammonium, ferrous iron, sulphide etc.) released from the sediment are oxidized^26^. Consideration of the latter allowed consistent estimates of the O_2_ budget for areal hypolimnetic mineralization (AHM) rates^27^. As a consequence, the concentration of O_2_ limits AHM in shallow lakes while the deposition of OM determines AHM in deep lakes. Sedimentary OM deposition during a former hypertrophic period, as experienced by almost all anthropogenically-influenced lakes in the 1970s, will delay recovery of the lake’s AHM rate^28^.

While the relationship between settling OM and hypolimnetic O_2_ consumption is conceptually well understood, the direct link between the annual P input and AHM is not as clear. In this study, we report the relationship between observed AHM rates and P supply from 21 European lakes of varying depth. The C:P ratios measured from settling material collected in sediment traps help to refine and better understand the elaborated relationship.

## Results and Discussion

### Decrease in oxygen consumption below a threshold of phosphorus supply

Biomass production in the epilimnion of a lake is limited by the total areal P supply (APS), i.e., the sum of total P present in the epilimnion at spring overturn plus the external supply of P available to phytoplankton (i.e. total dissolved P) during summer stratification (see methods section). Phosphorus input during the productive season can be significant and may even exceed the P amount already present in the epilimnion after spring overturn. This latter parameter is often incorrectly used as the key reference for P available for production, neglecting that especially in oligotrophic lakes or lakes with a short water residence time, both P present in the trophic layer and seasonal P input contribute substantially to primary production and subsequent hypolimnetic O_2_ consumption. The areal consumption of O_2_ in the hypolimnion (AHM) is caused by (i) mineralization of OM settling from the productive zone, (ii) O_2_ diffusion into the sediment, and (iii) reduced compounds diffusing from the sediment. The contribution of nitrate to the mineralization of OM is only ~3% of the O_2_ consumption rate^29^. The structure of the food web may also exert influence on O_2_ depletion^30^ but - due to missing data - was not considered here.

**Figure 1:**
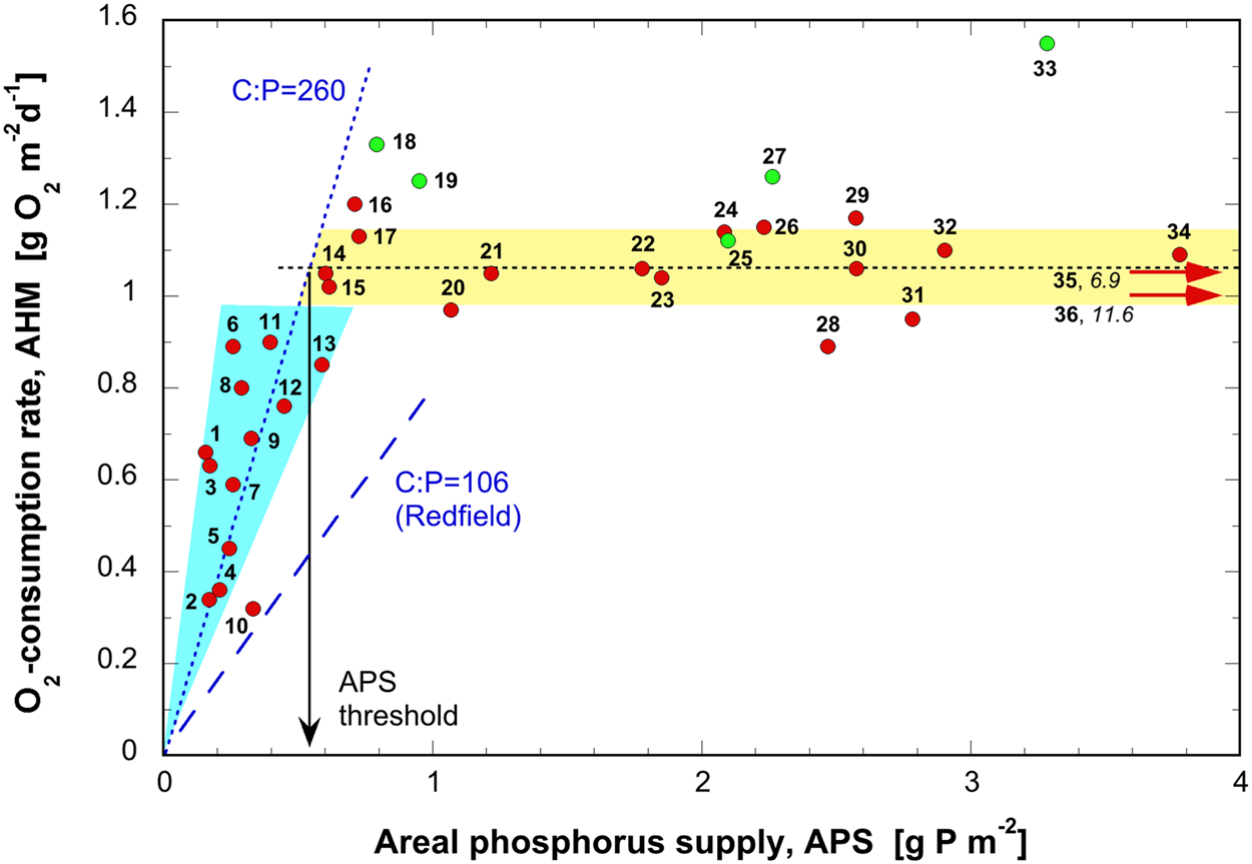
Covariation of areal hypolimnetic mineralization rate (AHM) with areal phosphorus supply per productive season (APS). The dotted black line in the teal sector represents a linear trend in data from lakes no. 1–13 with a slope of 1.96 ± 0.20 d^-1^. The horizontal dotted black line represents an average of lakes no. 14–36 (1.06 ± 0.08 g m^-2^ d^-1^, yellow bar). Green dots indicate artificially aerated lakes that were not included in calculations as their AHM was modified by technical measures. The intersection of 0.54 ± 0.06 g P m^-2^ per productive season indicates the observed APS threshold. At higher APS, the mineralization rate remains constant at 1.06 ± 0.08 g m^-2^ d^-1^ (yellow bar). Lakes are listed and described in Table S1. 1: Annecy (1995–2009), 2: Brienz (2000–2018), 3: Aegeri (2002–2012), 4: Sarnen (1994–2018), 5: Thun (2000–2017), 6: Neuchâtel (2000–2018), 7: Walensee (2007–2017), 8: Constance (2006–2016), 9: Aegeri (1975–1985), 10: Lucerne VB (1999–2018), 11: Hallwil (2010–2018), 12: Pfäffikon (2007–2017), 13: Maggiore (1988–2018), 14: Murten (2001–2017), 15: Sempach (2002–2018), 16: Geneva (2000–2010), 17: Neuchâtel (1963–1999), 18: Baldegg (2008–2018), 19: Hallwil (1998–2009), 20: Zürich (2000–2017), 21: Lucerne UB (1965–1974), 22: Lugano SB (1995–2006), 23: Greifensee (2000–2017), 24: Walensee (1976–1982), 25: Hallwil (1987–1994), 26: Geneva (1975–1985), 27: Sempach (1984–1992), 28: Zürich (1976–1990), 29: Constance (1973–1983), 30: Lucerne KT (1972–1982), 31: Sempach (1970–1983), 32: Lucerne VB (1965–1973), 33: Baldegg (1983–1992), 34: Lugano SB (1983–1990), 35: Pfäffikon (1963–1987), 36: Greifensee (1958–1968).

The relationship between the APS and AHM rates are summarized in Fig. 1. The results show that the AHM rates fall within a surprisingly narrow range of 1.06 ± 0.08 g O_2_ m^−2^ d^−1^ if APS exceeds 0.54 ± 0.06 g P m^−2^ (yellow area in Fig. 1). AHM only decreased when APS rates fell below the threshold of ~0.54 g P m^−2^. Below this threshold, AHM and APS rates are proportionally related (teal area in Fig. 1).

This finding agrees with the concept that at low APS rates, phytoplankton production, and thus the resulting OM sedimentation are limited by the availability of P. At elevated APS values, however, P no longer limits primary production and subsequently AHM. Phytoplankton then consume P in excess or leave it in solution. Thus, both primary production and AHM approach maximum values.

Outliers marked in green derive from Lakes Baldegg (BAL, 1983–1992, no. 33, and 2008–2018, no. 18), Sempach (SEM, 1984–1992, no. 27), and Hallwil (HAL, 1987–1994, no. 25, and 1998–2009, no. 19) all of which are aerated with pure O_2_ during the productive summer stratification^15^ to avoid anoxic hypolimnia. In eutrophic lakes, kinetic factors governing O_2_ flux into the sediment cause the benthic O_2_ consumption to increase in proportion to available [O_2_] and thereby generate the elevated AHM rates observed during summer^16,18^. Given these interventions in lake oxygen cycles, data from these lakes were not included in average calculations shown in Fig. 1.

The O_2_ consumption necessary for mineralization of organically bound carbon occurs at an observed ratio of 106:138 according to Eq. 1^31^.

1 $${({{\rm{CH}}}_{{\rm{2}}}{\rm{O}})}_{{\rm{106}}}{({{\rm{NH}}}_{{\rm{3}}})}_{{\rm{16}}}({{\rm{H}}}_{{\rm{3}}}{{\rm{PO}}}_{{\rm{4}}})+{{\rm{138O}}}_{{\rm{2}}}={{\rm{106CO}}}_{{\rm{2}}}+{{{\rm{16NO}}}_{{\rm{3}}}}^{-}+{{{\rm{HPO}}}_{{\rm{4}}}}^{{\rm{2}}-}+{{\rm{122H}}}_{{\rm{2}}}{\rm{O}}+{{\rm{18H}}}^{+}$$

Varying proportions of P and N do not significantly influence this ratio. Accordingly, an O_2_ consumption rate of 1.06 g m^−2^ d^−1^ shown in Fig. 1, corresponds to a mineralization rate of 56 g C m^−2^ over a stratified production period of six months. This value coincides with reported ranges of gross sedimentation from sediment traps and net sedimentation^18^. Only when the APS falls below the threshold of ~0.54 g P m^−2^ does the AHM rate decrease steeply in proportion to the P load. The transition of AHM from proportional increase with APS to the plateau is consistent with observations showing that net sedimentation of OM remains approximately constant when a threshold of P concentration is exceeded^32^. This suggests that phytoplankton can maintain maximum production under declining APS by incorporating less P relative to C up to a threshold of ~0.54 g P m^−2^.

### Continuously high production due to increasing C:P ratio

The Redfield stoichiometry (Eq. 1)^33,34^ suggests that in a P-limited system OM production or mineralization are strictly proportional to P uptake or release, respectively. Accordingly, the blue dashed line in Fig. 1 depicts the theoretical hypolimnetic O_2_ consumption (AHM) relative to APS (i.e. the P supply to the epilimnion, assuming the P:C:O_2_ = 1:106:138). Empirical data instead show that AHM decreased only if the available APS decreased below a threshold value of ~0.54 g P m^−2^ per productive season. The teal sector in Fig. 1 indicating a molar C:P ratio of about 260 (range of 240 to 300) instead of 106 suggests that microbial communities incorporate about 2.5 times more carbon per phosphorus than assumed in Eq. 1. Above the inflection point in the AHM-APS relationship, the AHM remains at a maximum level. This estimate is remarkably close to the concluding characterization of the first comprehensive lakes study by Vollenweider in 1971^1^ who stated that: *“a body of water is in danger with regard to its trophic level when its springtime concentration of assimilable phosphorus compounds exceeds 10 mg P m*^−3^ *and/or when the specific supply loading per unit area of lake reaches 0*.2*–0.5 g P m*^−*2*^ *per year*.”

### Sediment trap materials confirm increasing C:P ratios during re-oligotrophication

**Figure 2:**
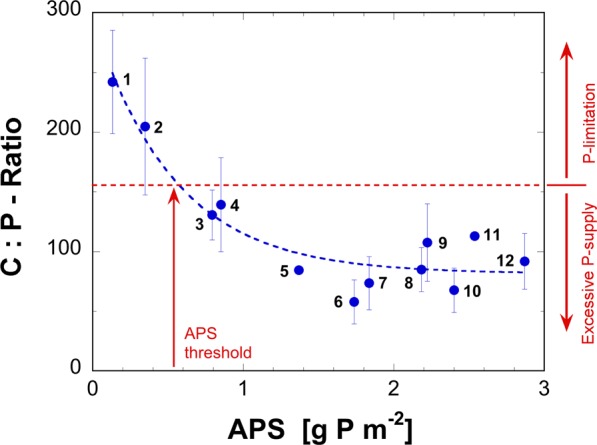
Molar ratios of C:P observed from settled material collected in sediment traps. Eight European lakes with widely varying APS were sampled and monitored over different time periods. 1: Lake Aegeri (2013–2014); 2: Lake Hallwil (2014–2016); 3: Lake Lucerne (1969); 4: Lake Baldegg (2013–2014); 5: Lake Zürich (1989); 6: Lake Sempach (1988–1993); 7: Lake Baldegg (1994–1996); 8: Lake Zürich (1984); 9: Greifensee (2002–2003); 10: Lake Sempach (1984–1987) 11: Lake Constance (1981–1982); 12: Greifensee (1989–1990). For further information and data sources, see supplemental Table S2.

Phytoplankton communities can apparently optimize carbon assimilation in case of diminished P availability during reoligotrophication. This manifests as increasing C:P ratios. The observed composition of settling particles collected by sediment traps in eight lakes – spanning a range of trophic states over 1- to 9-year observation periods - support the scenario described above. The C:P ratio of settling material increases with decreasing APS (Fig. 2) and confirms the ability of the phytoplankton community to cope with significantly less P than assumed by the Redfield ratio^35–38^. In contrast, under conditions of increasing P availability, plankton may take up P beyond its needs and store it, for example, as polyphosphates^39^. Combining the results presented in Figs. 1 and 2 leads to the conclusion that OM production is P-limited as long as i) APS ≤0.54 g P m^−2^ or ii) the C:P ratio exceeds ~155. In lakes with a high APS the C:P ratio of settling material reaches 80 ± 10 (see supplementary note 1). This value ranges clearly below the classical Redfield ratio of 106 and supports the idea of excess P storage by phytoplankton. However, ‘luxury storage’^40^ does not continuously increase with the availability of P but appears to stabilize at a maximum level. According to Fig. 1, primary production and thus AHM are P-limited as long as the APS does not exceed 0.54 g P m^−2^. Consequently, results presented in Fig. 2 suggest P limitation if the atomic C:P ratio of the settling material exceeds ~155. In summary, the wide scatter in the observed C:P ratios explains why in the case of strongly eutrophic lakes, a minor reduction of APS may not result in a corresponding lower AHM because phytoplankton starts only to starve for P and thus to decrease OM production, if its C:P ratio exceeds about 155.

### Delayed recovery due to sedimentary sequestration

Lakes with a mean hypolimnion depth z_hypo_ of less than 40 m that experienced an episode of eutrophic conditions with associated low hypolimnetic O_2_ concentrations in the past, accumulated large amounts of only partially degraded OM in the sediment^18^. The slow mineralization that occurs under anoxic conditions^28^ results in the formation of reduced compounds, e.g. methane and ammonium, which diffuse from the sediment to the water column^26^. Such fluxes of reduced compounds delay recovery from previously elevated AHM rates even once primary production has declined as a consequence of a lower APS. We consider 40 m as a critical hypolimnion depth because the O_2_ reservoir of such a lake is only about half way depleted by the end of the stratified season even if AHM rates reach maximum values (see supplementary note 2). In deep lakes (z_hypo_ > 40 m), this effect subsides due to their large hypolimnetic O_2_ reservoirs and extended settling distances, permitting nearly complete aerobic oxidation of the OM already while settling through the water column^25^. To sum up, in deep lakes only minor amounts of OM accumulate in the anoxic sediment and the consequently low benthic fluxes of reduced substances cannot significantly delay their recovery after reduction of APS below the threshold of 0.54 g P m^−2^.

### Implications for lake management

Water management agencies invest significant resources to lower P loads into natural water bodies. These expenditures are ultimately based on nutrient models seeking to minimize impact of the P load on the lakes’ O_2_ cycles. Data interpreted here reveal a distinct inflection point in the relationship between the areal hypolimnetic O_2_ consumption rate (AHM) and APS. Below an APS threshold of 0.54 g P m^−2^ per productive season, APS and AHM are proportionally related. Above this APS threshold, AHM rates take a constant value of 1.06 ± 0.08 g O_2_ m^−2^ d^−1^ independent of APS. Composition of settling particles collected by sediment traps affirms that the atomic C:P ratio of freshwater phytoplankton is highly variable and decreases strongly with increasing APS. Ratios of >155 indicate P limitation, whereas ratios <155 are characteristically observed in lakes that are excessively supplied with P. Thus, effective restoration of a lake suffering from too low hypolimnetic O_2_ concentration requires lowering its APS distinctly below the threshold value of 0.54 g P m^−2^. This refined understanding allows more accurate prediction of the minimum average hypolimnetic O_2_ concentrations observed at the end of summer stratification depending on a lake’s P supply, its mean hypolimnion depth and the hypolimnetic O_2_ concentration achieved by the end of the spring overturn ([O_2_]_0_).

### Estimation of the tolerable P supply to maintain a sufficient oxygen concentration

The effect of APS on AHM as depicted in Fig. 1 allows to estimate the tolerable APS (APS_tol_) preventing the hypolimnetic O_2_ concentration from decreasing below a desired minimum, [O_2_]_min_, by the end of the stratified period. As shown in Eq. 2, AHM_tol_ depends on the O_2_ concentration attained by the end of spring overturn, [O_2_]_0_, the tolerable minimum average hypolimnetic O_2_ concentration at the end of the stagnation period, [O_2_]_min_, the mean hypolimnion depth, z_hypo_, and the duration of the stratified period Δt_strat_.

2 $$AH{M}_{tol}=\frac{{z}_{hypo}}{\Delta {t}_{strat}}\times ({[{O}_{2}]}_{0}-{[{O}_{2}]}_{min})$$

Assuming [O_2_]_0_ to attain 10 g m^−3^ and the desired [O_2_]_min_ = 4 g m^−3^ (as requested e.g. by the Swiss water quality regulations) and Δt_strat_ to extend over 180 days yields

3 $$AH{M}_{tol}=0.033\times {z}_{hypo}$$

i.e., the observed AHM of 1.06 g O_2_ m^−2^ d^−1^ (Fig. 1) will most likely exceed the tolerable AHM in lakes with a mean hypolimnion depth shallower than ~32 m. This agrees well with the empirical finding that shallow lakes are more vulnerable to hypolimnetic anoxia than deep lakes. For lakes with shallower hypolimnia, Eq. 3 allows the estimation of AHM_tol_, and the corresponding tolerable APS can be derived from Fig. 1, using the slope of the initial correlation between AHM and APS of s = 1.96 d^−1^. APS_tol_ is thus

4 $$AP{S}_{tol}=\frac{AH{M}_{tol}}{s}$$

Combination of Eqs. 2 and 4 and replacement of APS_tol_ with the relationship for the estimation of APS (Eq. 6, methods section) and reorganization results in the formula for the calculation of the tolerable P load during the stratified period, ${\rm{L}}{{\rm{P}}}_{{\rm{t}}{\rm{o}}{\rm{l}}}^{{\rm{s}}{\rm{t}}{\rm{r}}{\rm{a}}{\rm{t}}}$:

5 $$L{P}_{tol}^{strat}[g{m}^{-2}]=\frac{{z}_{hypo}}{\Delta {t}_{strat}\times s}\times ({[{O}_{2}]}_{0}-{[{O}_{2}]}_{min})-[T{P}_{mix}]\times {z}_{epi}$$

where [TP_mix_] is the concentration after winter mixing and z_epi_ is the thickness of the epilimnion.

## Methods

### Lake monitoring data and AHM estimation

Monitoring data obtained from the Swiss Federal Office for the Environment (FOEN) database consisted of vertical water column profiles for oxygen, methane, ammonium, and nitrite. Measurements were performed according to standard methods^41^ after sampling various water layers at the lake’s deepest point at monthly or bi-weekly intervals (i.e., O_2_ measured by the Winkler method, ammonium and nitrite measured photometrically, and methane measured by gas chromatography of headspace gas). Contents of the corresponding lake epilimnia were estimated as volume-integrated mass assuming a mean epilimnion depth of 15 m^32^. Estimates of the hypolimnion O_2_ content were corrected for the accumulated reduced compounds methane, ammonium, and nitrite by subtracting the amount of the corresponding O_2_ equivalents necessary for their oxidation^18,27^. Areal hypolimnetic mineralization rates (AHM, [g O_2_ m^−2^ d^−1^]) within the water layer between 15 m and maximum depth were estimated as the difference between the maximum ‘corrected’ hypolimnion O_2_ content observed after spring overturn (February to April) and the minimum value observed at the end of summer stratification (late October/November) divided by the number of days of summer stratification. The inclusion of reduced compounds in addition to hypolimnetic O_2_ consumption increases the estimate of AHM by only a few percent. However, for hypertrophic lakes that accumulated high hypolimnetic concentrations of ammonium and methane by the end of the stratified period (such as e.g., Lake Pfäffikon, Türlersee, Greifensee), those reduced compounds can contribute up to 40% of AHM^27^.

### Estimation of the areal phosphorus supply APS

The APS, normalized to the area at 15 m water depth, supports the epilimnetic biomass production and thus the OM load to the hypolimnion below this area. As shown in Eq. 6, APS corresponds to the sum of the available P stock per area after spring overturn, plus the phytoplankton-available P supplied during summer stratification to the epilimnion volume laying above the hypolimnion^32^. Without further knowledge on the temporal distribution of phytoplankton-available P, we assume that the phytoplankton-available P imported during summer stagnation remained in the epilimnion and was fully available for primary production.

6 $$APS[gP{m}^{-2}]=([T{P}_{mix}]+\frac{LP\times 1yr}{{V}_{epi}}\times \frac{{Q}_{Apr-Sep}}{Q})\times {z}_{epi}$$

[TP_mix_] is the TP concentration of the lake after spring overturn in [g m^−3^]. LP is the annual load of total dissolved P in [g yr^−1^], V_epi_ is the volume of the epilimnion in [m^3^], Q_Apr-Sep_ and Q represent the water inflow for April to September and throughout the entire year in [m^3^], respectively, and z_epi_ the epilimnion depth of 15 m. Epilimnion depth as well as water inflow values derive from averages over the past ten years. Water discharge data were available from the Hydrological Service of the Swiss Federal Office for the Environment (FOEN)^42^ and the Office of Waste, Water, Energy, and Air of the Canton Zürich (AWEL)^43^.

The annual P load from the catchment, LP, was estimated using the extended Vollenweider steady-state mass balance model^33^ which results in:

7 $$LP={V}_{L}\times [T{P}_{mix}]\times (\sigma +\frac{\beta }{\tau })$$

where V_L_ is the lake volume [m^3^], and σ, β, and τ are the net sedimentation rate [yr^−1^], stratification factor [−], and hydraulic residence time [yr], respectively. Stratification factor β defined as the annual average ratio [TP_epi_]/[TP_mix_] was obtained from monitoring data. Net sedimentation rate σ was available in the form of direct sediment trap analyses from Lakes Sempach, Baldegg, Hallwil, and Sarnen or was estimated as 15 m/z_L_^32^, where z_L_ is the mean lake depth. For lakes that showed a distinct trend in their TP content over the considered time period (not at steady-state, as listed in Table S1), we corrected LP to consistently match the observed [TP]_mix_ concentrations.

### Sediment trap data

Sinking particles were collected either with cylindrical plexiglass tubes measuring 65 cm in length and 9.2 cm in diameter or using automated sequential traps^44^. These were installed 3 to 5 m above the lake bottom at its deepest point. Detailed information is provided in Supplementary Table 2. Samples were collected daily, biweekly, triweekly, or monthly, then freeze-dried and weighed. Analysis of total organic carbon and total nitrogen was performed using thermic combustion. TP was determined photometrically with the molybdenum blue method after digestion with potassium persulfate (K_2_S_2_O_8_) in an autoclave. Elemental ratios were averaged from samples collected between April and September of the years indicated in Fig. 2 captions.

## Supplementary information

Supplementary Information

## Data Availability

The authors declare the data supporting the findings of this study are available from the authors upon request.
