# Supplementary material for: Oxygen consumption in seasonally stratified lakes decreases only below a marginal phosphorus threshold
Source: Sci Rep. 2019 Dec 2;9:18054. doi: 10.1038/s41598-019-54486-3 (PMC6889406; doi:10.1038/s41598-019-54486-3)
Supplement: Supplementary file 1 — Supplementary Information [file 41598_2019_54486_MOESM1_ESM.pdf]

Supplemental information

Oxygen consumption in seasonally stratified lakes decreases only below a marginal phosphorus threshold

Beat Müller*, Thomas Steinsberger, Robert Schwefel, René Gächter, Michael Sturm, Alfred Wüest

Note 1:

Fitting function for increasing C:P ratio with declining APS in Figure 2:

An exponential function has been applied to fit experimental measurements of sediment trap material presented in Figure 2 (dashed blue line):

$$C\!:\!P - Ratio\ =\ a\ +\ b\ \times\ e^{-c\times APS} \qquad (1)$$

with a = 81 ± 10, b = 214 ± 30, c = 1.85 ± 0.54 m^2 $(g\ P)^{-1}$, and R = 0.95.

Note 2:

Shallow lakes and deep lakes

The areal hypolimnetic mineralization rate of eutrophic lakes increases in proportion to the lakes' mean hypolimnion depth and levels off between 30 m to 50 m depth[1]. Two processes contribute to the consumption of O_2: (i) the diffusive transport of bottom water O_2 across the diffusive boundary layer to the organic matter deposited at the sediment surface, and (ii) the oxidation of reduced compounds such as CH_4, NH_4^+, Fe(II), Mn((II) etc. in the bottom water released from the sediment. Mineralization of OM in the water column becomes apparent only in lakes of more than 100 m depth[2]. Process (i) is first order with respect to the concentration of O_2 and therefore, AHM increases in proportion to the concentration of O_2 in the lakes bottom waters. Therefore, lakes with a large hypolimnion depth that can store larger amounts of O_2 per area can keep up a higher AHM. Since AHM reaches a maximum due to the limited diffusion across the boundary layer, lakes with a critical mean hypolimnion depth do not run out of O_2 during the stratified season. Of course, this is only true for monomictic lakes.

A fully productive lake with a mean hypolimnion depth of ~40 m stores approximately 400 g O_2 m^{-2} after winter mixing, assuming a mean O_2 concentration of 10 g m^{-3}. At a maximum consumption rate of 1.1 g O_2 m^{-2} d^{-1} (Figure 1) during an average stratification period of 180 d, about 200 g O_2 m^{-2} or half of the O_2 stored in the hypolimnion is consumed. We therefore consider a mean hypolimnion depth of ~40 m as a critical value for lakes with respect to O_2 depletion. In a typical morphology of landscape, a hypolimnion depth of ~40 m corresponds to a lake of ~100 m maximum depth.

Table S-1: Lakes included in Figure 1. Numbers refer to the ones used in Figure 1 of the manuscript. Numbers behind names indicate the years of monitoring data used for averages. V_L: lake volume; A_L: lake surface; z_{max}: maximum depth; z_{mean}: mean lake depth; Q: water through-flow; τ: water residence time; V_H: hypolimnion volume; A_H: hypolimnion area; V_{epi}: epilimnion volume; β: stratification factor; σ: sedimentation rate; TP_{mix}: volume-weighted mean concentration of TP after spring mixing; Trend: Trend in TP_{mix} over the considered time period; LP_{ann}: averaged annual P-load; LP_s: P-load estimated from April to September (6 months); APS: areal P supply per productive season; AHM: areal hypolimnetic mineralization rate.

No	Lake	Years	V_L	A_L	z_{max}	Z_{mean}	Q	τ	V_H	A_H	V_{epi}	β	σ	TP_{mix}	Trend	LP_{ann}	LP_s	APS	AHM
			m^3	m^2	m	m	$m^3\ yr^{-1}$	yr	m^3	m^2	m^3	-	yr^{-1}	$mg\ m^{-3}$	$mg\ m^{-3}\ yr^{-1}$	$g\ yr^{-1}$	$g\ season^{-1}$	$g\ m^{-2}$	$g\ m^{-2}\ d^{-1}$
1	Annecy	1995-2009	1.03E+9	2.45E+7	65	41.9	2.56E+8	4.00	6.73E+8	2.22E+7	3.53E+8	1.10	0.35	5.5	0.0	3.51E+6	1.75E+6	0.157	0.66
2	Brienz	2000-2018	5.02E+9	2.98E+7	260	168.5	1.87E+9	2.69	4.59E+9	2.70E+7	4.29E+8	1.20	0.09	2.0	0.0	5.34E+6	4.00E+6	0.170	0.34
3	Aegeri	2002-2012	3.53E+8	7.30E+6	83	48.4	8.55E+7	4.13	2.51E+8	6.35E+6	1.02E+8	1.05	0.30	5.9	0.0	1.15E+6	5.77E+5	0.173	0.63
4	Sarnen	1994-2018	2.41E+8	7.15E+6	52	33.7	3.16E+8	0.76	1.44E+8	5.79E+6	1.44E+8	0.80	0.43	5.7	0.0	2.03E+6	1.19E+6	0.209	0.36
5	Thun	2000-2017	6.46E+9	4.79E+7	217	134.9	3.40E+9	1.90	5.78E+9	4.39E+7	6.94E+8	1.05	0.11	2.9	0.0	1.24E+7	9.33E+6	0.245	0.45
6	Neuchâtel	2000-2018	1.39E+10	2.18E+8	153	63.8	1.68E+9	8.25	1.11E+10	1.56E+8	2.67E+9	1.01	0.23	10.2	0.0	4.96E+7	1.89E+7	0.259	0.89
7	Walensee	2007-2017	2.51E+9	2.41E+7	145	104.1	1.73E+9	1.45	2.16E+9	2.28E+7	3.43E+8	1.10	0.14	3.1	0.0	6.99E+6	4.87E+6	0.259	0.59
8	Constance	2006-2016	4.78E+10	4.82E+8	254	99.2	1.10E+10	4.35	4.14E+10	4.03E+8	7.02E+9	1.00	0.15	7.6	0.0	1.37E+8	8.24E+7	0.291	0.80
9	Aegeri	1975-1985	3.53E+8	7.30E+6	83	48.4	8.55E+7	4.13	2.51E+8	6.35E+6	1.02E+8	1.01	0.30	11.9	-0.8	2.00E+6	1.00E+6	0.326	0.69
10	Lucerne VB	1999-2018	2.30E+9	2.20E+7	151	104.8	2.90E+9	0.79	1.98E+9	2.11E+7	3.23E+8	0.40	0.14	5.3	0.0	7.84E+6	5.45E+6	0.333	0.32
11	Hallwil	2010-2018	2.86E+8	9.95E+6	45	28.7	7.43E+7	3.85	1.51E+8	8.02E+6	1.34E+8	1.01	0.42	15.3	0.0	2.99E+6	1.50E+6	0.397	0.90
12	Pfäffikon	2007-2017	5.92E+7	3.20E+6	36	18.5	2.84E+7	2.09	1.89E+7	2.04E+6	4.03E+7	0.83	0.78	16.5	0.0	1.15E+6	5.39E+5	0.448	0.76
13	Maggiore	1988-2018	3.76E+10	2.13E+8	372	176.9	9.17E+9	4.10	3.45E+10	1.96E+8	3.06E+9	1.00	0.08	10.0	0.0	1.23E+8	8.58E+7	0.588	0.85
14	Murten	2001-2017	5.33E+8	2.30E+7	45	23.2	3.47E+8	1.54	2.58E+8	1.54E+7	2.75E+8	0.73	0.63	23.0	0.0	1.35E+7	4.70E+6	0.601	1.05
15	Sempach	2002-2018	6.42E+8	1.41E+7	86	45.5	3.66E+7	17.52	4.42E+8	1.24E+7	1.99E+8	0.92	0.30	25.0	0.0	6.75E+6	3.58E+6	0.617	1.02
16	Geneva	2000-2010	8.91E+10	5.82E+8	310	153.1	7.82E+9	11.40	8.08E+10	5.34E+8	8.33E+9	0.57	0.09	31.0	-1.7	2.48E+8	1.36E+8	0.711	1.20
17	Neuchâtel	1963-1999	1.39E+10	2.18E+8	153	63.8	1.68E+9	8.25	1.11E+10	1.56E+8	2.67E+9	0.75	0.23	29.7	0.0	1.31E+8	5.00E+7	0.727	1.13
18	Baldegg	2008-2018	1.74E+8	5.22E+6	66	33.4	3.74E+7	4.22	1.04E+8	4.10E+6	7.07E+7	1.05	0.68	25.2	-0.6	3.97E+6	1.96E+6	0.793	1.33
19	Hallwil	1998-2009	2.86E+8	9.95E+6	45	28.7	7.43E+7	3.85	1.51E+8	8.02E+6	1.34E+8	0.88	0.38	40.3	-2.7	6.21E+6	3.10E+6	0.952	1.25
20	Zürich	2000-2017	3.34E+9	6.66E+7	136	49.2	2.39E+9	1.40	2.45E+9	5.41E+7	8.96E+8	0.58	0.29	27.0	0.0	6.27E+7	3.67E+7	1.068	0.97
21	Lucerne UB	1965-1974	3.16E+9	2.20E+7	200	143.6	1.58E+9	2.00	2.84E+9	2.08E+7	3.20E+8	0.95	0.10	16.4	0.0	2.98E+7	2.07E+7	1.217	1.05
22	Lugano SB	1995-2006	1.12E+9	2.00E+7	95	56.1	8.01E+8	1.40	8.37E+8	1.81E+7	2.85E+8	0.57	0.26	51.2	-3.1	3.48E+7	1.92E+7	1.780	1.06
23	Greifensee	2000-2017	1.49E+8	8.45E+6	33	17.6	1.35E+8	1.10	4.46E+7	4.62E+6	1.04E+8	0.79	0.82	60.0	0.0	1.38E+7	6.60E+6	1.852	1.04
24	Walensee	1976-1982	2.51E+9	2.41E+7	145	104.1	1.73E+9	1.45	2.16E+9	2.28E+7	3.43E+8	1.10	0.14	25.9	-1.1	5.56E+7	3.87E+7	2.084	1.14
25	Hallwil	1987-1994	2.86E+8	9.95E+6	45	28.7	7.43E+7	3.85	1.51E+8	8.02E+6	1.34E+8	0.65	0.16	113	-11.2	7.31E+6	3.66E+6	2.097	1.12
26	Geneva	1975-1985	8.91E+10	5.82E+8	310	153.1	7.82E+9	11.40	8.08E+10	5.34E+8	8.33E+9	0.61	0.09	81.0	-0.5	1.03E+9	5.64E+8	2.230	1.15
27	Sempach	1984-1992	6.42E+8	1.41E+7	86	45.5	3.66E+7	17.52	4.42E+8	1.24E+7	1.99E+8	0.67	0.12	133	-8.6	6.61E+6	3.50E+6	2.264	1.26
28	Zürich	1976-1990	3.34E+9	6.66E+7	136	49.2	2.39E+9	1.40	2.45E+9	5.41E+7	8.96E+8	0.52	0.29	67.7	-3.2	1.37E+8	8.04E+7	2.468	0.89
29	Constance	1973-1983	4.78E+10	4.82E+8	254	99.2	1.10E+10	4.35	4.14E+10	4.03E+8	7.02E+9	0.50	0.15	82.9	0.0	1.04E+9	6.22E+8	2.572	1.17
30	Lucerne KT	1972-1982	1.52E+9	2.40E+7	112	63.3	3.46E+9	0.44	1.20E+9	2.40E+7	3.24E+8	0.57	0.23	28.7	0.0	6.65E+7	4.63E+7	2.574	1.06
31	Sempach	1970-1983	6.42E+8	1.41E+7	86	45.5	3.66E+7	17.52	4.42E+8	1.24E+7	1.99E+8	0.69	0.10	138	8.6	1.79E+7	9.46E+6	2.782	0.95
32	Lucerne VB	1965-1973	2.30E+9	2.20E+7	151	104.8	2.90E+9	0.79	1.98E+9	2.11E+7	3.23E+8	0.40	0.14	41.0	4.4	6.06E+7	4.22E+7	2.903	1.10
33	Baldegg	1983-1992	1.74E+8	5.22E+6	66	33.4	4.42E+7	4.22	1.04E+8	4.10E+6	7.07E+7	0.63	0.17	176	-20.3	6.14E+6	3.03E+6	3.283	1.55
34	Lugano SB	1983-1990	1.12E+9	2.00E+7	95	56.1	8.01E+8	1.40	8.37E+8	1.81E+7	2.85E+8	0.56	0.26	111	-8.4	7.26E+7	4.01E+7	3.777	1.09
35	Pfäffikon	1963-1987	5.92E+7	3.20E+6	36	18.5	2.84E+7	2.09	1.89E+7	2.04E+6	4.03E+7	0.76	0.78	265	-15.6	1.70E+7	7.95E+6	6.937	1.04
36	Greifensee	1958-1968	1.49E+8	8.45E+6	33	17.6	1.35E+8	1.10	4.46E+7	4.62E+6	1.04E+8	0.95	0.82	355	12.0	9.06E+7	4.34E+7	11.592	1.00

Table S-2: Sediment trap data shown in Figure 2. Element analyses from sediment trap material of eight lakes at different time intervals, and averages of TP_{mix} concentrations, APS and P loads in the corresponding periods. The load of algae-available P was calculated for the six months from April to September. The element ratios were averaged from trap samples collected between April and September.

**No.**	**Lake**	**sampling time**	**sampling depth**	**sampling interval**	**trap type**	**C:P**	**β**	**TP_{mix}**	**APS**	**Reference sed. traps**	**Reference P-loads**
		Years	m	d				mg P m^{-3}	g m^{-2}		
1	Aegeri	2014	62	14	Integrating	242	1.08	5.9	0.13	This study	Monitoring Canton Zug
2	Hallwil	2014-2016	30	28	Integrating	205	1.01	14	0.35	This study	Monitoring Canton Aargau
3	Lucerne	1969	60	14	Integrating	131	0.68	18	0.79	*3*	*4*
4	Baldegg	2013-2014	62	14	integrating	139	1.10	27	0.85	This study	Monitoring Canton Lucerne
5	Zürich	1989	130	21	Integrating	84	0.52	46	1.37	*5*	*6*
6	Sempach	1988-1993	81	14	Integrating	58	0.66	111	1.74	This study	Monitoring Canton Lucerne
7	Baldegg	1994-1996	64	1	sequential	74	0.64	88	1.84	This study	Monitoring Canton Lucerne
8	Zürich	1984	130	21	Integrating	85	0.51	61	2.18	*7*	*6*
9	Greifensee	2002-2003	31	28	Integrating	108	0.67	85	2.22	This study	Monitoring Canton Zürich
10	Sempach	1984-1987	81	14	Integrating	68	0.69	156	2.40	This study	Monitoring Canton Lucerne
11	Constance	1981-1982	div.	21	Integrating	113	0.60	83	2.54	*8*	*9*
12	Greifensee	1989-1990	28	21	Integrating	92	0.68	135	2.87	*10, 11*	Monitoring Canton Zürich

References:

1. Müller, B., Bryant, L. D., Matzinger, A. & Wüest, A. Hypolimnetic oxygen depletion in eutrophic lakes. *Environ. Sci. Technol.* **46,** 9964-9971 (2012). Doi: 10.1021/es301422r.
2. Schwefel, R. et al. Using small-scale measurements to estimate hypolimnetic oxygen depletion in a deep lake. *Limnol. Oceanogr.* **63,** S54–S67 (2018). Doi: 10.1002/lno.10723.
3. Bloesch, J. Sedimentation und Phosphorhaushalt im Vierwaldstättersee (Horwer Bucht) und im Rotsee. *Schweiz. Z. Hydrol*. **36/1**, 71-186 (1974).
4. Müller, B. Abschätzung der mittleren Phosphor-Konzentration im Vierwaldstättersee für verschiedene Eintragsfrachten. Expert report for the Canton of Lucerne (2017).
5. Wieland, E., Liebemann, P., Bollhalder, S., Lück, A. & Santschi, P. H. Composition and transport of settling particles in Lake Zürich: relative importance of vertical and lateral pathways. *Aquatic Sci.* **63**, 123-149, 2001.
6. Gammeter, S., Forster, R. & Zimmermann, U. Limnologische Untersuchung des Zürichsees 1972-1996. Wasserversorgung Zürich Qualitätsüberwachung, Zürich (1997).
7. Sigg, L., Sturm, M. & Kistler, D. Vertical transport of heavy metals by settling particles in Lake Zürich. *Limnol. Oceanogr.* **32/1**, 112-130 (1987).
8. Sigg, L. Metal transfer mechanisms in lakes. In: *Chemical Processes in Lakes*, ed Stumm, W., p. 283-307 (1985).
9. IGKB, Report Nr. 54, 2002, Tolerierbare Phosphor-Fracht des Bodensee-Obersees. Ber. Int. Gewässerschutzkomm. Bodensee: 54, 2002, ISSN 1011-1263 (2002).
10. Sigg, L., Kuhn, A., Xue, H. Kiefer, E. & Kister, D. Cycles of trace-elements (copper and zinc) in a eutrophic lake - Role of speciation and sedimentation. In: Huang, C. P., O'Melia, C. R., & Morgan, J. J. (Eds.), *Advances in Chemistry Series: Vol. 244. Aquatic Chemistry: Interfacial and Interspecies Processes,* p. 177-194 (1995).
11. Peel, K., Weiss, D. & Sigg, L. Zinc isotope composition of settling particles as a proxy for biogeochemical processes in lakes: Insights from the eutrophic Lake Greifen, Switzerland. *Limnol. Oceanogr.* **54/5**, 1699-1708 (2009).
